# SARS-CoV-2 infection in microglia and its sequelae: What do we know so far?

**DOI:** 10.1016/j.bbih.2024.100888

**Published:** 2024-10-12

**Authors:** Echo Yongqi Luo, Raymond Chuen-Chung Chang, Javier Gilbert-Jaramillo

**Affiliations:** a Laboratory of Neurodegenerative Diseases, School of Biomedical Sciences, LKS Faculty of Medicine, The University of Hong Kong, Hong Kong SAR, China; b State Key Laboratory of Brain and Cognitive Sciences, The University of Hong Kong, Hong Kong SAR, China; c James & Lillian Martin Centre, Sir William Dunn School of Pathology, University of Oxford, South Parks Road, Oxford, OX1 3RE, UK

**Keywords:** SARS-CoV-2, COVID-19, Microglia, CNS, Neuroinflammation

## Abstract

Severe acute respiratory syndrome coronavirus 2 (SARS-CoV-2) caused the COVID-19 pandemic. After the success of therapeutics and worldwide vaccination, the long-term sequelae of SARS-CoV-2 infections are yet to be determined. Common symptoms of COVID-19 include the loss of taste and smell, suggesting SARS-CoV-2 infection has a potentially detrimental effect on neurons within the olfactory/taste pathways, with direct access to the central nervous system (CNS). This could explain the detection of SARS-CoV-2 antigens in the brains of COVID-19 patients. Different viruses display neurotropism that causes impaired neurodevelopment and/or neurodegeneration. Hence, it is plausible that COVID-19-associated neuropathologies are directly driven by SARS-CoV-2 infection in the CNS. Microglia, resident immune cells of the brain, are constantly under investigation as their surveillance role has been suggested to act as a friend or a foe impacting the progression of neurological disorders. Herein, we review the current literature suggesting microglia potentially been a susceptible target by SARS-CoV-2 virions and their role in viral dissemination within the CNS. Particular attention is given to the different experimental models and their translational potential.

## Highlights

• SARS-CoV-2 may infect CNS, affecting microglia and brain cells.
• Neuroinflammation from SARS-CoV-2 could lead to long COVID memory issues.
• Microglia's role in COVID-19 neuropathologies remains unclear.
• Targeting microglia-mediated inflammation may prevent COVID-19 neuropathies.

## 1 Background

Coronavirus disease 2019 (COVID-19) is a sequela of viral infection by severe acute respiratory syndrome coronavirus 2 (SARS-CoV-2) and dysregulated host immune response (V'kovski et al., 2021). Although SARS-CoV-2 is a respiratory virus, it has been suggested to be associated with a myriad of neuropsychiatric complications during acute and post-acute stages (Chou et al., 2021), up to 6 months after the disease onset. Neuropsychiatric complications related to SARS-CoV-2 infections are termed COVID-19-associated neuropathologies and may include cognitive deficits (Quan et al., 2023; Taquet et al., 2022a), sensory disturbances (Trott et al., 2022), and sleep disorders (Alimoradi et al., 2021). Of relevance, a two-year retrospective cohort study on 1.28 million patients found a persistently increased risk of psychosis, cognitive impairment, dementia, and epilepsy (Taquet et al., 2022b).

During the COVID-19 pandemic, viral mutations increased the infectivity of new strains with implications for transmissibility and hospitalisation (Markov et al., 2023). Some of these strains, termed delta and omicron, have been predominantly linked to the epidemiology of COVID-19-associated neuropathologies (Taquet et al., 2022b). Taquet et al., 2022a, Taquet et al., 2022b suggested that vaccination against SARS-CoV-2 may protect against secondary outcomes such as seizures and psychotic disorders but not psychiatric comorbidities and long-COVID presentations (Taquet et al., 2022a). However, little is known about waning vaccine-mediated immunity over time and immune escape by emerging variants (Chi et al., 2022). This highlights the importance of understanding the potential mechanisms of how SARS-CoV-2 virions can reach the central nervous system (CNS) and whether SARS-CoV-2 can productively infect cells within the CNS.

Among the CNS, microglia are the primary line of defence against infiltrating particles including viruses (Waltl and Kalinke, 2022). Microglia are the resident immune cells of the CNS, where their surveillance function and response to changes in the microenvironment in health and disease (i.e., infection and injury) are crucial for homeostasis (Prinz et al., 2019). In their resting state, microglia extend fine processes that dynamically scan the surrounding neural tissue, ready to respond to any disturbances (Nimmerjahn et al., 2005). Upon encountering pathogens, including viruses like SARS-CoV-2, microglial functions may include phagocytosis of circulating virions and/or infected cells, secretion of cytokines/chemokines, and, recruitment of peripheral immune cells (Waltl and Kalinke, 2022). Direct SARS-CoV-2 infection of microglia or microglial reactivity to neighbouring infected cells may lead to neuroinflammation. Unresolved neuroinflammation may consequently damage the CNS contributing to neurodegenerative and neuropsychiatric disorders (Kwon and Koh, 2020; Najjar et al., 2013). Hence, elucidating whether microglial cells are susceptible to SARS-CoV-2 infection would signify a step forward in understanding potential mechanisms for COVID-19-associated neuropathologies.

**Fig. 1:**
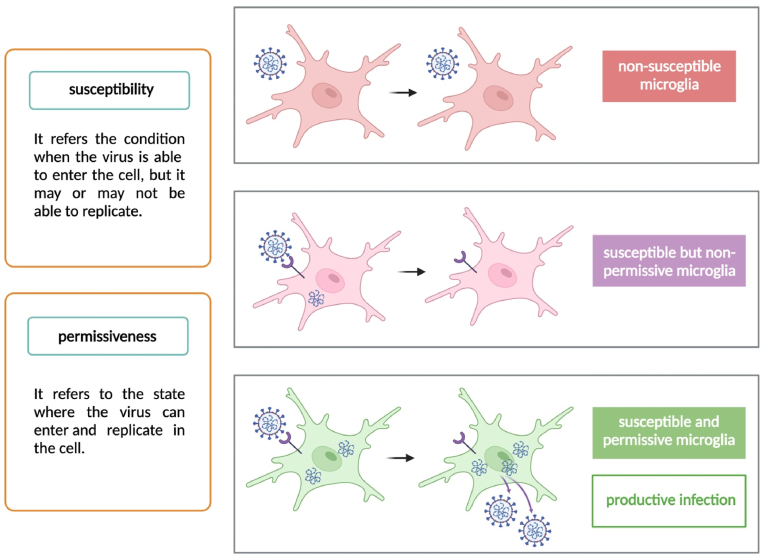
| Susceptibility and permissiveness of microglia to SARS-CoV-2. Based on the definitions of susceptibility and permissiveness (shown on the left), productive infection requires the microglia to be both susceptible and permissive to SARS-CoV-2. From top to bottom, the schematics illustrates the scenarios of non-susceptibility, abortive infection and productive infection. The figure was created with BioRender.com.

**Fig. 2:**
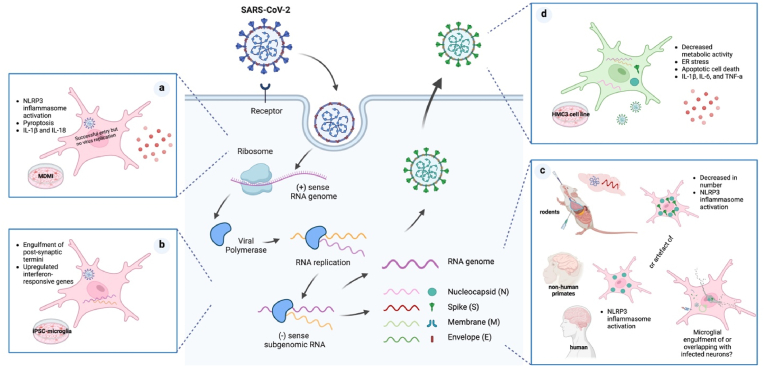
(Summary Diagram) | Putative SARS-CoV-2 lifecycle in microglia. The life cycle of SARS-CoV-2 involves the virus binding to host cell receptors, entering the cell through endocytosis or membrane fusion, releasing its single-stranded positive-sense RNA genome, which is translated and replicated into double-stranded RNA intermediates, followed by the production of new viral RNA and proteins, assembling virions, and finally exiting the host cells to infect new cells. Different viral markers in microglia demonstrate varied susceptibility, permissiveness, and cellular responses to SARS-CoV-2 in different human microglia models, rodent models, non-human primates, and post-mortem brains from COVID-19 patients. (a) In monocyte-derived microglia models, only the entry of viruses were detected; (b) In iPSC-derived microglia, double-stranded RNA, a hallmark of viral replication, were found but insufficient to prove efficient infection; (c) In rodent and human brain tissues, only single-stranded spike RNA and spike/ nucleocapsid proteins were colocalized, the possibility of microglia engulfing infected neutrons with viral particles could not be excluded; (d) Only *in vitro* human microglia cell line (HMC3) allowed the full lifecycle of SARS-CoV-2, with the key evidence of both double-stranded RNA and increased extracellular virions indicating effective infection. Shown in bullet points are the sequelae of the exposure to SARS-CoV-2 in different experimental models. The figure was created with BioRender.com.

**Fig. 3:**
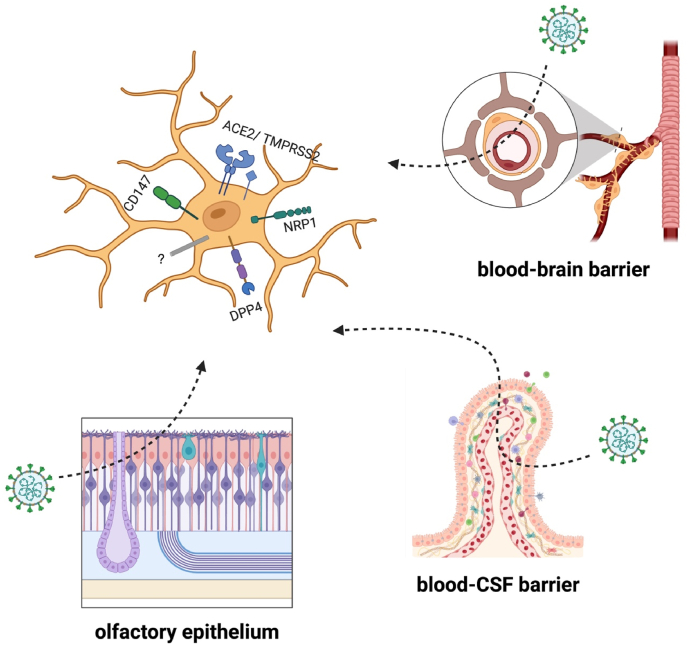
| Surface molecules related to SARS-CoV-2 infection of microglia and possible routes of brain invasion. Neuropilin-1 (NRP-1) interacts with the spike at the furin cleavage site, crucial for the viral entry. Dipeptidyl Peptidase-4 (DPP4) and Cluster of Differentiation 147 (CD147, also known as basigin or extracellular matrix metalloproteinase inducer) may act as a co-receptor of ACE2 for the spike, enhancing viral attachment and entry, especially in tissues where ACE2 expression is limited. More noncanonical surface molecules related to viral entry are yet to be determined. SARS-CoV-2 may reach the brain via the blood-brain barrier, blood-CSF barrier, and olfactory nerves. The figure was created with BioRender.com.

**Fig. 4:**
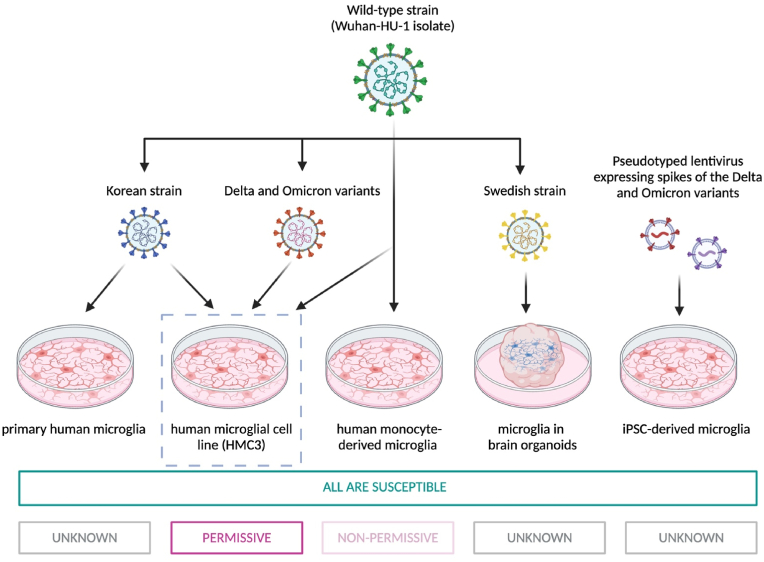
| Experimental diversity in terms of virus strains and human microglia models. Only the HMC3 cell line was found to allow productive infection by wild-type strain, Korean strain, Delta variants, and Omicron variants. Different virus strains/variants mostly differ in the spikes for entry. All microglia models permit the entry of all strains but differ in permissiveness potentially suggesting different microglia models differ in the post-entry response to SARS-CoV-2 strains/variants. The figure was created with BioRender.com.

**Fig. 5:**
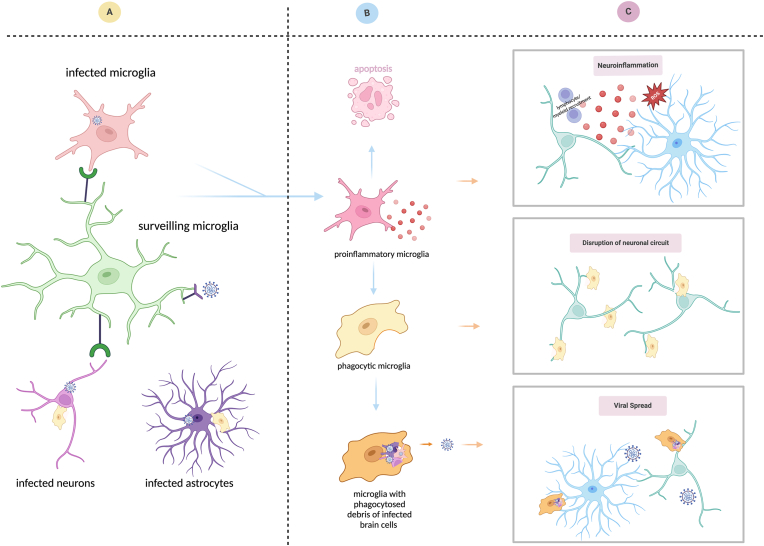
| The sequelae of SARS-CoV-2 infection in microglia. (A) Microglia can be activated by exposure to SARS-CoV-2, infected microglia, and other infected brain cells such as neurons and astrocytes. (B) Infected microglia or activated microglia exhibit proinflammatory and phagocytic phenotypes. (C) Microglia with engulfed debris from infected brain cells are motile, potentially spreading viruses to various brain regions. Chronic activation of microglia may result in aberrant neuroinflammation detrimental to neurons, and excessive synaptic pruning that disrupts neuronal circuits and exacerbates preexisting neuropathologies. The figure was created with BioRender.com.

**Fig. 6:**
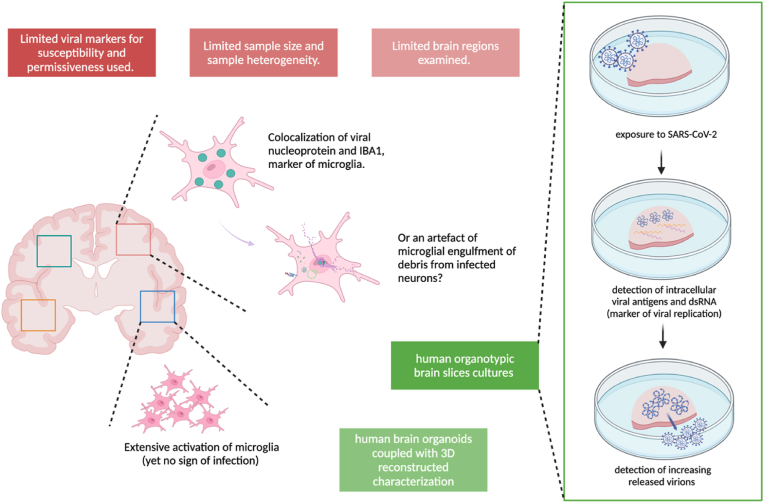
| Limitations and future perspectives for current evidence from post-mortem COVID-19 patients' brains. Current research limitations (red boxes) to determine microglial infection by SARS-CoV-2 include a) insufficient viral markers such as dsRNA and increasingly released virions and, b) discrepancies in detecting viral markers colocalized with microglia potentially due to small sample sizes, limited screening of brain regions, and sample heterogeneity. Henceforth, future research (green boxes) using *in vitro* systems may be needed to elucidate single mechanisms of infection that could support current findings. For example, using human organotypic brain slice cultures or brain organoids exposed to SARS-CoV-2 allows resesarchers to track the dynamics of SARS-CoV-2 replication within microglia, including the process of virion release. In these systems, microglia may respond to viral infection more closely mimicking in vivo conditions given the more native brain environment, compared to monocultures. The figure was created with BioRender.com.

In this review, we explore the potential routes by which SARS-CoV-2 may invade the CNS, focusing on the viral susceptibility and permissiveness of microglia (Fig. 1). We synthesize current evidence from both post-mortem human and non-human brains to examine the likelihood of productive microglial infection (Fig. 2 & Table 1). Additionally, we consider how factors such as surface molecules (Fig. 3) and experimental diversity in virus strains and models (Fig. 4) contribute to our understanding of the SARS-CoV-2 infection and its sequelae in microglia (Fig. 5). By appreciating the complexity and specificity of these interactions, we aim to highlight the limitations of current research and suggest future perspectives (Fig. 6), particularly in the context of data derived from post-mortem COVID-19 patients, as we discuss in the concluding sections.

**Table 1:** | Summary of putative SARS-CoV-2 infection of different human and non-human microglia models, rodent models, non-human primates, and post-mortem brains from COVID-19 patients. Susceptibility is evidenced by the intracellular detection of viral antigens (genetic materials or proteins) while permissiveness is determined by the increased detection of infectious particles over time. Only HMC3 showed sufficient evidence for both susceptibility and permissiveness to SARS-CoV-2. Abbreviation: N = nucleocapsid; S = spike protein; NP = nucleoprotein; dsRNA = double-stranded RNA; IBA1 = ionised calcium-binding adaptor molecule 1; NLRP3 = a central nucleotide-binding and oligomerisation domain (NOD) + a C-terminal leucine-rich repeat domain (LRR) + an amino-terminal pyrin domain (PYD); NF-κB = nuclear factor kappa B; IL = interleukin; ER = endoplasmic reticulum; iPSC = induced pluripotent stem cells; RT-qPCR = reverse transcription-quantitative polymerase chain reaction; IHC = immunohistochemistry; WB = western blotting; NA = not available.

		SARS-CoV-2 strains	SARS-CoV-2 Infection	Mecha nisms	Susceptibility	Permissiveness	Microglial responses	Reference
Human Microglia Model	Human monocyte-derived microglia (MDMi)	Wild-type strain/Wuhan-HU-1 isolate (GISAID accession no. EPI_ISL_ 407896)	Successful entry but no replication.	Spike-ACE2 interaction	YES. Pseudo-virus entry assay and reporter-virus replication assay indicate successful entry but failed replication.	NO. Confirmed by growth kinetics (no virions released to supernatant).	NLRP3 inflammasome activation through NF-κB signalling. Generation of IL-1β and IL-18. Initiation of pyroptosis.	Albornoz et al. (2023)
	Human microglial cell line (HMC3)	Wild-type strain (GISAID accession no. EPI_ISL_407073), Alpha variants (GISAID accession no. EPI_ISL_693401), Beta variants (lineage B.1.351), Delta variants (GISAID accession no. EPI_ISL_1731019), Eta variants (lineage B.1.525), Omicron variants (lineage BA.1)	Productive infection by WT, Delta and Omicron variants.	May be mediated by ACE2. TMPRSS2, CD147, and NRP1.	YES. Confirmed by intracellular detection of N protein with IHC.	YES. Confirmed by the cytopathic effect with MTS assay and the xCELLigence assay (Cell Proliferation/Viability Assay).	Decreased metabolic activity in response to WT and Delta infection.	Proust et al. (2023)
		Korean strain (GISAID accession no. MW466791)	Successful entry and life cycle completion.	NA.	YES. Confirmed by (1) detection of N1 vRNA with RT-qPCR; (2) colocalisation of NP and S/dsRNA with IHC; (3) presence of NP with WB.	YES. Confirmed by the cytopathic effect under the microscope and with focus forming assay.	M1-like proinflammatory responses (production of IL-1β, IL-6, and TNF-a). ER stress. Apoptotic cell death (both intrinsic and extrinsic death receptor-mediated).	Jeong et al. (2022)
	Primary human microglia (PHM)/iPSC-microglia		Successful entry.		YES. Confirmed by detection of N1 vRNA with RT-qPCR.	NA.	NA.	
	iPSC-derived microglia	SARS-CoV-2_S (B.1.617.2/Delta)(B.1.1.529/Omicron) pseudotyped lentivirus	Successful entry.	May be DPP4-mediated.	YES. Confirmed by reporter expression.	NA.	NA.	Kase et al. (2023)
	Microglia innately developed in undirected brain organoids	Swedish strain (GenBank: MT093571.1)	Successful entry and replication.	NA.	YES. Confirmed by colocalisation of dsRNA and IBA1 with IHC.	NA.	Upregulation of interferon-responsive genes. Promoted engulfment of post-synaptic termini.	Samudyata et al. (2022)
Rodents	K18-ACE2 mice	Intranasally injected Korean strain (GISAID accession no. MW466791)	Successful brain invasion.	Spike-ACE2 interaction	Unclear. vRNA copies occur in the brain and colocalisation of spike and microglia were observed.	NA. Reduced body weight following intranasal administration was observed.	Decreased in number.	Jeong et al. (2022)
		Intranasally injected Wuhan strain (GISAID accession no. EPI_ISL_ 407896)			Unclear. Colocalisation of microglia and viral capsid were observed.		Extensive microglial activation and NLRP3 inflammasome upregulation.	Albornoz et al. (2023)
		Intraperitoneally injected USA-WA1/2020 strain			Unclear. Spike mRNA increased in the brain and vRNA were distributed in various brain regions.		Microgliosis.	Olivarria et al. (2022)
Non-human primates	Rhesus macaques	Intratracheally injected USA-WA1/2020 strain	Successful brain invasion.	Spike-ACE2 interaction	Unclear. Colocalisation of NP and IBA1 were observed.	NA.	Extensive neuronal synaptic pruning and myelin degradation. Microglial proliferation and translocation.	Beckman et al. (2022)
	Rhesus macaques and Cynomolgus macaques	Intratracheally or intranasally injected BetaCoV/BavPat1/2020 strain	Viral RNA present but no evidence of active virus replication.	NA.	NA.	NA.	Microglial activation was observed.	Philippens et al. (2022)
	Rhesus macaques and African green monkeys	Multi-route mucosal or aerosol exposure to USA-WA1/2020 strain	Limited to brain vasculature (endothelial cells).	NA.	NA.	NA.	Microglial activation as indicated by upregulated Iba-1 and HLA-DR and enlarged volumes. Increased proximity to blood vessels.	Rutkai et al. (2022)
	Rhesus monkeys	Intranasally or intracranially injected SARS-CoV-2 (strain unknown).	Successful brain invasion.	Spike-ACE2 interaction	Unclear. Colocalisation of NP and IBA1 were observed.	NA.	Upregulated RPS11, RPS13, and RPL13 but downregulated ND3, ATP6, and COX3 in hippocampal microglia.	Jiao et al. (2021)
Post-mortem COVID brains	Deceased COVID-19 patients	NA.	Brain invasion was found.	Spike-ACE2 interaction	Unclear. Colocalisation of NP and Iba-1 were identified with IHC.	NA.	Microglial activation as indicated by NLRP3 inflammasome detection.	(Cama et al., 2021; Poloni et al., 2021; Zhang et al., 2023; Schwabenland et al., 2021)
			Brain invasion was not found.	NA.	No SARS-CoV-2 transcripts detected in the brain/microglia.	NO.	However, microglia were distinctly activated.	Fullard et al. (2021)
		Alpha & Beta variants						Grant et al. (2024)

## 2 SARS-CoV-2: Biology and route of entry into the CNS

SARS-CoV-2, a non-segmented single-stranded positive sense (+ss) RNA virus, belongs to the family of coronaviruses. Other members of this family include pandemic viruses (i.e. MERS-CoV) and non-pandemic viruses (i.e. HCoV-229E) (Lim et al., 2016). These viruses are among the most respiratory infectious for humans (Perlman, 2020). SARS-CoV-2 genome encompasses a 5′ methylated cap and 3′ poly-A tail to function directly as message RNA, which encodes four structural proteins: spike (S), envelope (E), membrane (M) and nucleocapsid (N) (Yang and Rao, 2021). The two open reading frames (ORF) 1a and 1b encode 15–16 non-structural proteins that compose the viral replication and transcription complex (V'kovski et al., 2021). This is necessary for the integrity maintenance of the coronavirus genome. Lowly conserved accessory proteins, encoded by specific ORFs, play a crucial role in the diversity of modulatory immune responses and pathogenicity (Redondo et al., 2021). For instance, ORF3b is an interferon antagonist (Konno et al., 2020), and ORF8 binds to MHC-I and mediates its lysosomal degradation for immune evasion (Zhang et al., 2021).

Once in the respiratory tract, SARS-CoV-2 infect susceptible cells via angiotensin-converting enzyme 2 (ACE2) receptors. The viral S proteins are responsible for this process in which the functional surface-exposed part S1 binds to ACE2, followed by the viral fusion to the cellular membrane mediated by the functional transmembrane part S2 domain (Jackson et al., 2022). Besides, the virus utilises the cell-surface serine proteases TMPRSS2 for priming and entry by proteolytic cleavage of S proteins, a process assisted by the endosomal cysteine proteases cathepsin B and L (Wettstein et al., 2022). This highlights essential factors required for productive SARS-CoV-2 infection, factors that are differentially expressed among different cell types (Jackson et al., 2022). In the brain, ACE2 is minimally expressed across different regions, with neurons in the substantia nigra being the most enriched (Chen et al., 2021). Like other immune cells (e.g., monocytes, macrophages, and T lymphocytes), microglia express low levels of ACE2 (Yang et al., 2021; Iadecola et al., 2020). Nonetheless, it is plausible that SARS-CoV-2 infects microglia by binding to other receptors such as DPP4/CD147 that could mediate viral entry (Kase et al., 2023; Li et al., 2020; Radzikowska et al., 2020; Raj et al., 2013; Wang et al., 2020) and/or exploit noncanonical entry modes (Andrews et al., 2022; Crunfli et al., 2022).

Multiple routes of entry of SARS-CoV-2 into the brain involve crossing the blood-brain barrier (BBB) which could be achieved via several mechanisms, such as diffusion, direct infection of endothelial cells (Krasemann et al., 2022; Wenzel et al., 2021; Yamada et al., 2024; Zhang et al., 2021), cell-mediated transport, or transcytosis (Ayala-Nunez and Gaudin, 2020; Krasemann et al., 2022). Furthermore, SARS-CoV-2 can compromise BBB by downregulating tight junctions (Yamada et al., 2024) or inducing the death of brain endothelial cells (Wenzel et al., 2021). However, recent evidence indicates that SARS-CoV-2 primarily targets epithelial cells of the blood-cerebrospinal fluid barrier rather than endothelial cells or pericytes of the BBB (Stüdle et al., 2023), suggesting alternative pathways for CNS entry. This process is further complicated by the role of systemic inflammation (Greene et al., 2024), a hallmark of SARS-CoV-2 infection, which increases the permeability of brain barriers, making the CNS more vulnerable, not only to viral invasion but also to systemic toxins and stressors (Greene et al., 2024; Knox et al., 2022). Increased permeability also facilitates the infiltration of circulating leukocytes potentially carrying virions into the CNS. This promotes neuroinflammation and serves as a virus-exploited “trojan horse” mechanism of virion release and infection of brain cells (Gilbert-Jaramillo et al., 2019). In addition, SARS-CoV-2 may exhibit tropism for non-microglial brain cells such as neurons or astrocytes; the latter is a potential potent driver of neuroimmune signalling and microglial dysregulation as observed in some neuropsychiatric conditions (Kwon and Koh, 2020). Thus, a potential neuroinflammatory scenario (Krasemann et al., 2022) may facilitate SARS-CoV-2 infection of microglia via a) direct exposure to high virion load, b) direct exposure to virions within a proinflammatory environment, c) indirect exposure via engulfment of infected brain cells, and, d) engulfment of transsynaptic traffic which could ultimately facilitate SARS-CoV-2 spread across the brain due to the normal motility and migration of microglial cells.

Moreover, the anatomy of the respiratory tract may suggest a direct route of entry into the CNS. Hence, the latent interest in developing intranasal immunisation (Deimel et al., 2022; Formica et al., 2022). Anosmia in COVID-19 patients may be linked to a disruption in the nasal epithelium and/or communication between olfactory neurons and mitral cells (Brann et al., 2020; de Melo et al., 2021). This could be due to productive infection of SARS-CoV-2 to neuronal tissue, which may promote microglial infection by direct exposure to high viral loads upon phagocytosis. Therefore, there is the possibility that SARS-CoV-2 virions reach the CNS to encounter several cell types potentially permissive to infection dependent or independent of ACE2-mediated cell entry (see Fig. 3). To date, SARS-CoV-2 infection in brain cells is mainly linked to astrocytes (Crunfli et al., 2022) via ACE2 independent entry (Andrews et al., 2022) with discrepant data on microglial infection (Andrews et al., 2022; Cama et al., 2021; Crunfli et al., 2022).

## 3 Human microglia models and SARS-CoV-2 infection

In vitro models of human microglia have one consensus intention; to simulate microglia in vivo. Three main functional characteristics can be used to determine how well in vivo microglia are recapitulated in these cell sources: (1) cell surface markers; (2) inflammatory responses; and (3) phagocytotic capacity (McMillan et al., 2023). Nevertheless, heterogeneity at the transcriptomic level between different sources of microglia, such as immortalised primary microglia (imMG/HMC3), adult post-mortem microglia (pmMGa), primary fetal microglia (MGf), and induced microglia-like cells from peripheral blood mononuclear cells (PBMCs; iMG), remains an issue for disease modelling (Sellgren et al., 2017).

HMC3 cells were established through the polyomavirus simian virus 40 (SV40) large T antigen-dependent immortalization of primary human microglial cells derived from 8-to-10-week-old embryos. HMC3 cultures comprise a mixture of parenchymal microglia and other myeloid populations (Dello Russo et al., 2018). HMC3 grows rapidly and retains most phenotypical and morphological characteristics of the primary microglial cells, except for a higher percentage of CD68-positive cells, lower responsiveness to LPS stimuli, and, lower phagocytic activity (Dello Russo et al., 2018). The latter is potentially mediated by a downregulated panel of genes (P2ry12, C1qa, Gas6, Mertk, Gpr34, and Pros1) involved in phagocytosis and migration (Butovsky et al., 2014). The lower phagocytic activity of HMC3 may relate to reduced capacity to a) internalise and destroy viral particles, b) clear infected cells and c) present antigens and recruit adaptive immune cells such as cytotoxic CD8^+^ T cells, resulting in prolonged infection and sustained inflammatory response as an attempt to counter the ongoing viral presence. This combination of factors, along with other considerations detailed in Section 4.1., may help explain why HMC3 is the only model that allows productive infection by SARS-CoV-2. Results obtained from HMC3 cells, especially those concerning viral clearance and the resolution of inflammation, should be interpreted with caution, as they may overestimate the permissiveness and inflammatory responses of microglia in vivo.

MGf and iMG share similar transcriptomes suggesting that, in contrast to pmMGa, these cells may mimic fetal stage microglia. Other microglial models involve monocyte-derived microglia and human pluripotent stem cell-derived microglia (hiPSC-derived microglia) (Hanger et al., 2020). hiPSC-derived microglia are a robust model due to the homogenous populations obtained after differentiation, yet, donor-to-donor variation is to be considered (Hanger et al., 2020). Moreover, hiPSC-derived microglia often undergo cell death after prolonged times under culture, thus limiting experiments to the short-time assessment of the kinetics of viral infection (Jeong et al., 2022). Thus, in summary, the choice of microglia models to study SARS-CoV-2 infection should serve the research objective and may require special attention for translational purposes.

## 4 Discussion: Preclinical evidence of SARS-CoV-2 infection of microglia and its sequelae

Productive infection of SARS-CoV-2 in microglia remains unclear. In vivo results in rodent models, mostly hACE2-K18 transgenic mice (Albornoz et al., 2023; Olivarria et al., 2022; Jeong et al., 2022), showed productive infection of SARS-CoV-2 in neuronal cells yet not in microglia. Microglia from non-human primates (NHPs) is closely related to that of humans thus offering a platform to overcome limitations in brain studies such as tissue processing after long postmortem times. Postmortem staining of NHP brains corroborated SARS-CoV-2 productive infection in neuronal cells yet lacked sufficient evidence to suggest this phenomenon occurs in microglia (Beckman et al., 2022; Jiao et al., 2021; Philippens et al., 2022). Moreover, NHP infected brains show a highly inflammatory profile (Beckman et al., 2022; Jiao et al., 2021; Philippens et al., 2022; Rutkai et al., 2022) that correlates with a permeable BBB with infiltration of systemic immune reactive cells which could potentially alter the microenvironment and susceptibility of microglia to SARS-CoV-2 infection as previously discussed.

Furthermore, among various human models of microglia, productive infection of SASRS-CoV-2 has been shown exclusively in the HMC3 (Albornoz et al., 2023; Jeong et al., 2022; Samudyata et al., 2022). Thus, these discrepant results may have arisen from the heterogeneity in experimental design primarily concerning a) the choice of microglia models, b) SARS-CoV-2 strains, and c) the methodology of infection.

### 4.1 Experimental diversity: human microglia models and SARS-CoV-2 strains

Albornoz et al. (2023) found that SARS-CoV-2 (ancestral Wuhan strain) could bind to and enter, yet not infect, monocyte-derived microglia (MDMi) (Albornoz et al., 2023). Interestingly, the entry of SARS-CoV-2 in MDMi induces NF-κB-mediated NLRP3 inflammasome activation (Albornoz et al., 2023). Conversely, Jeong et al. (2022) found productive SARS-CoV-2 infection in HMC3 and no activation of the NLRP3 inflammasome when challenging these cells with the SARS-CoV-2 Korean strain (Jeong et al., 2022). Thus, these discrepancies could be attributed to selective viral mutations originated during SARS-CoV-2 *in vitro* propagation (Amicone et al., 2022) or to the cellular models exhibiting differential susceptibility to SARS-CoV-2 infection. Thus, sequencing to verify for selective mutations may be required to better conclude the infectivity potential of SARS-CoV-2 in microglia (see Fig. 4).

The selection of cellular models to reduce variability *in vitro* is crucial for the accurate interpretation of results. Cell model-specific properties lead to varied outcomes of viral infection. For instance, Wuhan strains efficiently infect HMC3 (Proust et al., 2023) but not MDMi (Albornoz et al., 2023), probably due to immortalization-induced changes in antiviral responses (detailed in the next paragraph) and/or origin differences. The former from embryonic spinal cord and cortical cells. The latter from bone marrow-derived monocytes. Moreover, hiPSC-derived microglia, expressing a significant level of dipeptidyl peptidase 4 (DPP4, a recognised receptor for MERS-CoV), were susceptible to spike-expressing pseudotype Delta, and Omicron viruses yet, their permissiveness required further investigation (Kase et al., 2023), particularly as spike-independent mechanisms of entry could be elicited.

Furthermore, it remains discursive whether the efficient infection is accounted for by the SV40 immortalization-induced alterations in HMC3 or the virus-specific strategies to evade microglial defenses or both. HMC3 are immortalised primary human fetal microglia through the introduction of viral SV40 large T antigen (LTA) that inhibits tumour suppressors (e.g., p53). The tumour suppressor pathways crosstalk with antiviral defence, apoptosis, and pro-inflammatory pathways (Hare et al., 2016). Thus, the removal of certain tumour suppressors may silence antiviral genes that, in the case of HMC3, may promote their susceptibility to SARS-CoV-2 productive infection (Hare et al., 2016). This could explain recent findings (Proust et al., 2023) in HMC3 expressing relevant receptors i.e., ACE2, TMPRSS2, CD147, and NRP1, where no productive infection by some strains of SARS-CoV-2, but to Wuhan, Delta, and Omicron was reported.

Albeit HMC3 is the only proven human microglial cell line in which SARS-CoV-2 productive infection is observed yet not by all strains and all neurotropic viruses, suggesting virus-specific immune evasion. (Jeong et al., 2022; Proust et al., 2023). For example, while primary microglia are known to be the main CNS target for HIV-1 (Wallet et al., 2019), HMC3 cells show reduced HIV replication despite producing similar cytokine and chemokine profiles (Dello Russo et al., 2018; Peudenier et al., 1991; Rawat and Spector, 2017). Besides, it is resistant to murine norovirus MV1 (Cox et al., 2009) and some coronaviruses like the huCV-229E subgroup (Arbour et al., 1999). Thus, immune evasion strategies could involve e.g., induction of TRIM21 and downregulating STAT1 to facilitate viral replication as induced by the neurotropic Japanese encephalitis virus (JEV; Manocha et al., 2014).

Productive infection requires the host cell to be susceptible (allowing viral binding and entry) and permissive (permitting completion of viral cycles) to SARS-CoV-2 virions. Thus, the detection of viral antigens or viral RNA only reflects susceptibility (Bauer et al., 2022). In HMC3, perinuclear colocalisation of viral nucleoprotein (NP) and dsRNA has been shown as a productive infection (Jeong et al., 2022). However, this may reflect artefacts of immunofluorescent techniques when the analysis is conducted in 2D and could potentially explain the insignificant changes in released virions over time (Jeong et al., 2022). In addition, small changes in released virions over time may be due to potential microglial detection of dsRNA, when reaching a specific intracellular abundance, via e.g., cGAS-STING pathway as shown in other viruses such as Zika viruses (Zheng et al., 2018).

The experimental variability stemming from the choice of cell models, virus strains, and experimental conditions presents significant challenges in translating findings to accurately reflect in vivo microglial responses to SARS-CoV-2 infection. This stresses the importance of standardized experimental protocols, cross-validation with multiple cell lines with extensive genetic and phenotypic profiling, and detailed transparent methodological documentation.

### 4.2 SARS-CoV-2 effects on microglial homeostasis

Exposure of microglia to SARS-CoV-2 over six days suggests a shift from an inflammatory to a phagocytic state (Jeong et al., 2022) (see Fig. 5). At day three, microglia exhibited increased ER stress and antiviral responses; the latter mainly involving type I interferons (IFN) and cytokine-mediated signalling whilst, at day six, activated pathways indicate phagocytosis and apoptosis (Jeong et al., 2022). These differential cellular responses over time may potentially be due to a synergistic outcome of heterogeneous populations of infected and non-infected neighbouring cells within the same culture as demonstrated for other viruses (Gilbert-Jaramillo et al., 2023). Nonetheless, strong evidence proposes neuroinflammation as a feature of SARS-CoV-2 infection in the CNS. To exemplify, in cortical-blood vessel assembloids, SARS-CoV-2-induced cell death mostly occurred in TUNEL^+^ HIF1α^+^ NP^−^ cells adjacent to NP^+^ cells, suggesting the viruses could indirectly kill cells by inducing a locally hypoxic environment (Kong et al., 2023) whilst in undirected brain organoids containing microglia, single-cell RNA sequencing revealed that SARS-CoV-2 activated microglial interferon signaling and upregulated phagocytosis-related pathways (Samudyata et al., 2022). The latter, however, remains under investigation as opposing results from colocalisation of immunofluorescent probes have been reported (Samudyata et al., 2022). Thus, further investigation into potential mechanisms of microglial clearance of SARS-CoV-2 virions is required (see Fig. 5).

Furthermore, microglial homeostasis involves a close interaction between immune and bioenergetic pathways. Viruses are known entities that suppress/activate several components of these pathways for advantageous purposes in both immune and non-immune cells (Gilbert-Jaramillo et al., 2019, 2023). SARS-CoV-2 transcriptionally rewires microglial homeostasis by increments in components of unfolded protein response, ER stress, proteasomal degradation, autophagy and downregulation of mitochondrial metabolism (Samudyata et al., 2022). Thus, further investigation on potential increments in bioenergetic-immune crosstalk upon SARS-CoV-2 cellular detection may elucidate the subsequent cascade of cellular alterations. Lastly, the extent to which transcriptomic alterations in microglia are induced by SARS-CoV-2 needs further investigation as current single timepoint transcriptomic findings may solely reflect the “snapshot” during sample acquisition.

### 4.3 Clinical relevance of transgenic animal models

Most in vivo models of SARS-CoV-2 infection in microglia comprise the use of hACE2-K18 transgenic mice. This model was developed by the introduction of transgene copies of hACE2 into chromosome 2 of the mouse. This is a big difference compared to humans as the hACE2 gene is located on the X chromosome (Winkler et al., 2020) which potentially underlies the sex differences (Muñoz-Fontela et al., 2020). Moreover, it remains unclear whether human and transgenic mice brains express comparable tissue-specific patterns of entry receptors and their impact on SARS-CoV-2 neurotropism. In addition, variable survival post intracranial exposure to SARS-CoV-2 may reduce sample sizes and thus the study of long-term neurological responses to SARS-CoV-2 infection (Muñoz-Fontela et al., 2020). Thus, more robust in vivo models e.g., wild-type Syrian hamsters (Muñoz-Fontela et al., 2020) and/or the insertion of endogenous promoter-driven co-receptors such as human TMPRSS2, in addition to hACE2, may be required. To this end, a growing body of literature has emerged using NHPs which resemble adult human brains to a greater level than rodent models (Beckman et al., 2022; Jiao et al., 2021; Philippens et al., 2022; Rutkai et al., 2022). However, current findings strongly evidencing neuroinflammatory environments do not answer whether this inflammation is caused by microglial cells and if so, whether it is due to a) being productively infected, b) responding to detected internalised virions, c) crosstalk and phagocytosis of infected cells or d) modulated by the inflammatory microenvironment.

## 5 Microglia in the SARS-CoV-2 infected CNS: friend or foe?

The effect of inhibiting microglial recognition and reactivity to SARS-CoV-2 infection is yet to be elucidated (see Fig. 5). However, understanding the response mechanisms of phagocytes and their consequences on the microenvironment may open different venues for therapeutics against uncontrolled inflammation. Extensive activation of microglia, yet no signs of infection were observed in post-mortem COVID-19 brains (Emmi et al., 2023; Poloni et al., 2021; Schwabenland et al., 2021; Zhang et al., 2023). This is potentially associated with infiltration of proinflammatory CD8^+^ T cells (Schwabenland et al., 2021). A feature of extensive microglial activation is their increased phagocytosis which, in the case of SARS-CoV-2 infection in the CNS, might act as a “double-sword” – they might prune potential viral transsynaptic transmission or they might initiate excess synaptic pruning disrupting neuronal circuits which ultimately results in memory loss, cognitive decline, or neuropsychiatric disturbances (Kwon and Koh, 2020).

Moreover, microglial infection by SARS-CoV-2 and their role in viral spread within the CNS remains unclear (see Fig. 5). According to Jeong et al. (2022), SARS-CoV-2 infection in K18-hACE2 mice reduced the microglial number, possibly due to upregulated apoptosis and proinflammatory activation whilst Olivarria et al. (2022) showed that SARS-CoV-2 infection in K18-hACE2 mice resulted in microgliosis. These partially discrepant results may be influenced by methodological artefacts such as the intrinsic differences in data collection between flow cytometry and immunohistochemistry (Olivarria et al., 2022; Jeong et al., 2022). Nevertheless, neuroinflammation, in which microglia may play a pivotal role, is a consensus feature across murine (Olivarria et al., 2022; Jeong et al., 2022) and NHP (Beckman et al., 2022; Rutkai et al., 2022) models of SARS-CoV-2 infection. Thus, *in vitro* studies modulating microglial activation may reveal distinct features in i.e., acute versus chronic SARS-CoV-2 infection which could help target neuroinflammation within the CNS during infection and in the context of long COVID.

## 6 Limitations and future perspectives

Current evidence showing SARS-CoV-2 infection in the CNS remains unclear, with potential routes of entry still under debate. Immunostaining-based studies showing SARS-CoV-2 antigens in microglia of post-mortem human brains (Cama et al., 2021; Schwabenland et al., 2021) may reflect the presence of viral particles at a particular time point yet not productive infection (See Fig. 6). Discrepant results from colocalisation analysis of SARS-CoV-2 viral proteins with specific markers of microglial cells in brain samples (Cama et al., 2021; Crunfli et al., 2022; Emmi et al., 2023; Schwabenland et al., 2021; Zhang et al., 2023) may solely reflect issues related to small sample sizes and sample heterogeneity. Moreover, whilst some of the existing animal models for COVID-19 are limited in mimicking severe or long-lasting clinical features and multi-system comorbidities of humans (Fan et al., 2022) others recapitulate clinical features yet sample processing of brain tissues still imposes difficulties in understanding the kinetics of SARS-CoV-2 infection in the CNS. Thus, more investigation using *in vitro* systems may help elucidate specific pathogenic mechanisms that could explain the observations of in vivo models and patients (See Fig. 6).

Single-cell omics studies on SARS-CoV-2 infection of human brains bring valuable insights into the molecular and cellular responses among distinct neuroimmune cell populations. Signs of microglial activation were consistently found despite the absence of viral antigens in brain tissues (Fullard et al., 2021; Grant et al., 2024), suggesting a robust neuroinflammatory response even without direct viral infection. The increased proportion of stromal cells, monocytes, and macrophages in the choroid plexus of severe COVID-19 patients likely reflects a defensive response against viral infection, which may explain the lack of detectable SARS-CoV-2 in the brain despite the heightened activation of cortical microglia (Fullard et al., 2021). Nonetheless, peripheral immune cells in the blood circulation (Cheong et al., 2023) and cerebrospinal fluid (Hu et al., 2024) could carry a durable epigenetic memory of coronavirus infection contributing to altered immune functions and long-COVID symptoms. This raises complex questions about the role of microglia and circulating leukocytes in the neurological symptoms associated with COVID-19. Thus, future studies on SARS-CoV-2 and microglia may require the coupling of advanced imaging techniques with omics approaches to delineate whether microglia are directly infected by the virus or if their activation is a secondary response to systemic inflammation, and how this may correlate with CNS dysfunction. This provide implications for the development of therapeutics, such as targeted enhancement of microglial mechanisms to prevent infection or dampening microglial activation to decrease neuronal damage.

## 7 Conclusions

There is the possibility of SARS-CoV-2 virions reaching the CNS and infecting brain cells, yet current data on viral infection in microglial cells is not conclusive and thus the role of microglia in potential COVID-19-associated neuropathologies remains unclear. Nevertheless, a hallmark of SARS-CoV-2 systemic infection is a cascade of neuroinflammatory responses that could lead to aberrant microglial activities which subsequently render individuals at a higher risk of developing memory issues, currently englobed under long COVID and neuropsychiatric complications. Thus, more efforts in understanding the differential profile of the different CNS cell types when exposed to SARS-CoV-2 is required as, for example, targeting potential microglia-mediated neuroinflammation in some COVID-19 patients might prevent the development of COVID-19-associated neuropathologies (Albornoz et al., 2023; Cama et al., 2021; Jeong et al., 2022; Zhang et al., 2023; Beckman et al., 2022; Schwabenland et al., 2021; Poloni et al., 2021).

## CRediT authorship contribution statement

**Echo Yongqi Luo:** Writing – review & editing, Writing – original draft, Visualization, Conceptualization. **Raymond Chuen-Chung Chang:** Writing – review & editing, Supervision. **Javier Gilbert-Jaramillo:** Writing – review & editing, Writing – original draft, Supervision.

## Research ethics

Not applicable.

## Funding

This research did not receive any specific grant from funding agencies in the public, commercial, or not-for-profit sectors.

## Declaration of competing interest

The authors declare that they have no competing interests.

## Data Availability

No data was used for the research described in the article.
